# Supplementary material for: Biocatalytic Method for Producing an Affinity Resin for the Isolation of Immunoglobulins
Source: Biomolecules. 2024 Jul 14;14(7):849. doi: 10.3390/biom14070849 (PMC11274487; doi:10.3390/biom14070849)
Supplement: Supplementary file 1 [file biomolecules-14-00849-s001.zip › biomolecules-3080047-supplementary.pdf]

Biocatalytic Method for Producing an Affinity Resin for the Isolation of Immunoglobulins

Mikhail N. Tereshin [1,2,†], Tatiana D. Melikhova [3,†], Barbara Z. Eletskaya [3,*], Elena A. Ivanova [4], Lyudmila V. Onoprienko [3], Dmitry A. Makarov [2], Mikhail V. Razumikhin [5], Igor V. Myagkikh [3], Igor P. Fabrichniy [4] and Vasiliy N. Stepanenko [1,2]

1 Lomonosov Institute of Fine Chemical Technologies, MIREA—Russian Technological University, Vernadskogo Pr. 86, 119571 Moscow, Russia; misha060596@yandex.ru (M.N.T.); stepanenko_vasy@mail.ru (V.N.S.)
2 Federal State Autonomous Educational Institution of Higher Education I.M. Sechenov First Moscow State Medical University of the Ministry of Health of the Russian Federation; 8-2 Trubetskaya Str., 119991 Moscow, Russia; youngchemist@mail.ru
3 Shemyakin-Ovchinnikov Institute of Bioorganic Chemistry, Russian Academy of Sciences, Miklukho-Maklaya St. 16/10, 117437 Moscow, Russia; tdm-63@yandex.ru (T.D.M.); onolv@mail.ru (L.V.O.); myagkikh@ibch.ru (I.V.M.)
4 International Biotechnology Center "Generium" LLC, Vladimirskaya st. 14, 601125 Volginsky, Russia; lenchem98@ya.ru (E.A.I.); fabri@ibcgenerium.ru (I.P.F.)
5 GreenVan LLC, Vodnikov Street, 16/2, of. 6, 125362 Moscow, Russia; razumikhin.m@gmail.com
* Correspondence: fraubarusya@gmail.com
† These authors contributed equally to the work.

Content **Page number**

Content	Page number
1. Preparation of polyglycine spacers for cross-linked agarose modification<br>1.1 Materials and reagents	SI-2
1.2 General procedure for the synthesis of succinimide ether of diglycine (Boc-Gly-Gly-OSu)	SI-2
1.3 General procedure for synthesis of Boc-protected polyglicine (n =3, 4, 5)	SI-2
2. Spectra of Boc-protected polyglicine (n =3, 4, 5) compounds	SI-3
2.1 ^{1}H NMR Boc-Gly$_3$-OH	SI-3
2.2 ^{1}H NMR Boc-Gly$_4$-OH	SI-4
2.3 ^{1}H NMR Boc-Gly$_5$-OH	SI-5

1. Preparation of polyglycine spacers for cross-linked agarose modification

1.1 Materials and reagents

N-oxysuccinimide, N, N'-dicyclohexylcarbodiimide (Aldrich), HBTU (Alfa Aesar), HOBT (Angene, China), mercaptoethanol, ethanolamine, 1,4-diamonobutane, Boc-Gly-Gly, glycine, diglycine, triglycine (Sigma Aldrich), sodium hydroxide (PanReac ApBCplichem, USA) , sodium acetate (Serva GmbH, Germany), tris(hydroxymethyl)aminomethane (Tris-HCl) (Searchbio, China), sodium bicarbonate (Reachim, Russia), sodium chloride (Labochem international), potassium chloride (Reachim, Russia), sodium hydrophosphate (Sigma), potassium dihydrogen phosphate (Merck). All solvents were used without additional cleaning. Trifluoroacetic acid, ethyldiisopropylamine, isopropanol, ethyl acetate, diethyl ether, tetrahydrofuran dimethylformamide (Chimmed, Russia).

NMR spectra were recorded on a Bruker Avance II 700 spectrometer (Bruker BioSpin, Rheinstetten, Germany) in DMSO-d_6 at 30 °C. Chemical shifts in ppm (δ) were measured relative to the residual solvent signals as internal standards (2.50). The coupling constants (J) were measured in Hz.

Liquid chromatography mass spectrometry was performed using an Agilent 6210 TOF LC–MS system (Agilent Technologies, Santa Clara, CA, USA). Detection was performed spectrophotometrically at 214 nm and using the positive (ES+) mode of electrospray ionization.

DBC_{10} estimation was carried out using a GE AKTA Pure chromatography system (GE Healthcare Bio-Sciences, Uppsala, Sweden) equipped with UNICORN software 7.3 SP1 (GE Healthcare Bio-Sciences, Uppsala, Sweden). The determination of protein concentration was carried out on a UV/visible spectrophotometer, Ultrospec-1000 (Amersham Pharmacia Biotech, Cambridge, UK).

1.2 General procedure for the synthesis of succinimide ether of diglycine (Boc-Gly-Gly-OSu)

Scheme S1-1. Scheme of succinimide ether synthesis

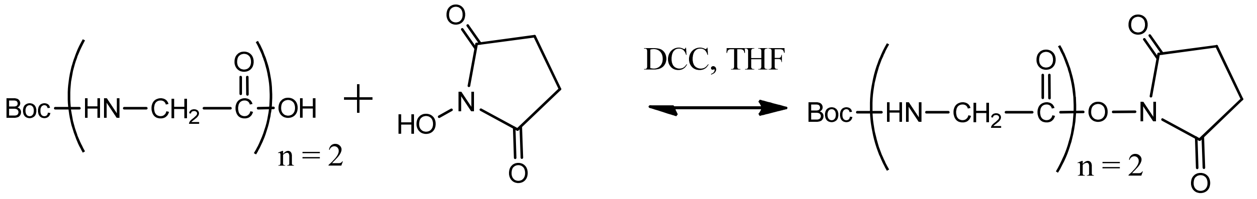

A solution of Boc-Gly$_2$-OH (3.48 g, 0.015 mol) and N-hydroxysuccinimide (2.3 g, 0.02 mol) was prepared in 7 ml of tetrahydrofuran (THF) and cooled to −5 °C. A solution of N,N'-dicyclohexylcarbodiimide (3.09 g, 0.015 mol) in THF was added to the cooled solution with vigorous stirring and cooled to −5 °C. The reaction mixture was stirred for 1 hour at a temperature from −5 to 0 °C, and then for 18 hours at room temperature. The reaction was monitored using thin-layer chromatography of Silica Gel 60 F 254 TLC plate (Merck, Germany) in a chloroform:ethanol (9:1) system. The cooling procedure was repeated and N,N'-dicyclohexylcarbodiimide (1.5 g, 0.007 mol) was added. The resulting precipitate was filtered and washed with THF. The resulting filtrate was concentrated on a rotary evaporator, crystallized from a mixture of diethyl ether : ethyl acetate (1:1), and dried in vacuum over phosphorus pentoxide. The yield of Boc-Gly$_2$-OSu is 3.56 g (72%).

1.3 General procedure for synthesis of Boc-protected polyglicine (n =3, 4, 5)

A sample of glycine (0.9 g, 0.012 mol), or diglycine (1.58 g, 0.012 mol) or triglycine (2.27 g, 0.012 mol) was dissolved in a saturated sodium bicarbonate solution in water (2 ml) and 1N NaOH (1 ml). A 10 ml solution of Boc-Gly-Gly-OSu (3.56 g, 0.011 mol) in a dioxane-water mixture (2:1) was added to the resulting solution. The mixture was stirred for 18 hours at room temperature. The course of the reaction was analyzed using thin-layer chromatography on a Silica Gel 60 F 254 TLC plate (Merck, Germany). The plate was placed in acetonitrile and water mixture (4:1), with 1 of sodium acetate added per 100 ml of the solvent system. To remove impurities, the reaction mixture was loaded on a column with Dowex 50W X8 hydrogen resin from Serva (Heidelberg, Germany). The solvent from the final product was removed using a rotary evaporator Buchi R-20 (BUCHI, Flawil, Switzerland). The residue was dissolved in isopropanol and lyophilized under ~~in~~ vacuum. The yield of Boc-Gly$_3$-OH is 1.80 g (52%), Boc-Gly$_4$-OH is 2.19 g (49%), Boc-Gly$_5$-OH is 2.6 g (45%)

2. Spectra of Boc-protected polyglicine (n =3, 4, 5) compounds

2.1 Boc-Gly$_3$-OH. HRMS, m/z: calculated for $C_{11}H_{19}N_3O_6$ $[M + H]^+$ 290.2935, found $[M + H]^+$ 290.2940.^{1}H NMR (700 MHz, DMSO) δ 8.12 (t, *J* = 5.66 Hz, 1H, NH), 8.04 (t, *J* = 5.14 Hz, 0.9H, NH), 6.98 (t, *J* = 5.75Hz, 0.9H, NH-Boc), 3.76 (d, *J* = 5.93 Hz, 2H, CH_2), 3.74 (d, *J* = 5.84 Hz, 2H, CH_2), 3.58 (d, *J* = 5.84 Hz, 1.9H, CH_2), 3.31 (br.s, 1H, OH), 1.39 (s, 9H, Boc) ppm. ^{13}C NMR (176 MHz, DMSO) δ 171.56 and 170.10 and 169.44 ($CH_2\underline{C}O$), 156.29 ($\underline{C}(O)O(CH_3)_3$), 78.59 ($(C(O)\underline{C}(CH_3)_3$), 43.79 and 42.28 and 41.39 ($\underline{C}H_2CO$), 28.67 ($C(O)O(\underline{C}H_3)_3$) ppm.

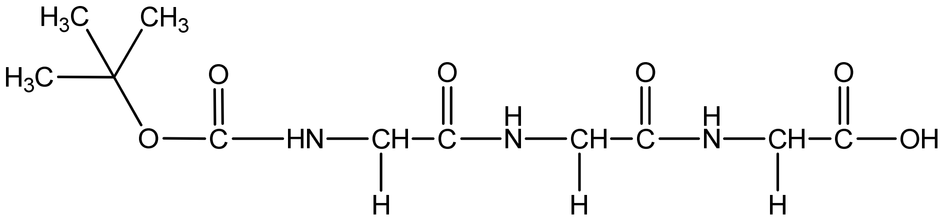

Chemical Formula: $C_{11}H_{19}N_3O_6$
Molecular Weight: 289,29

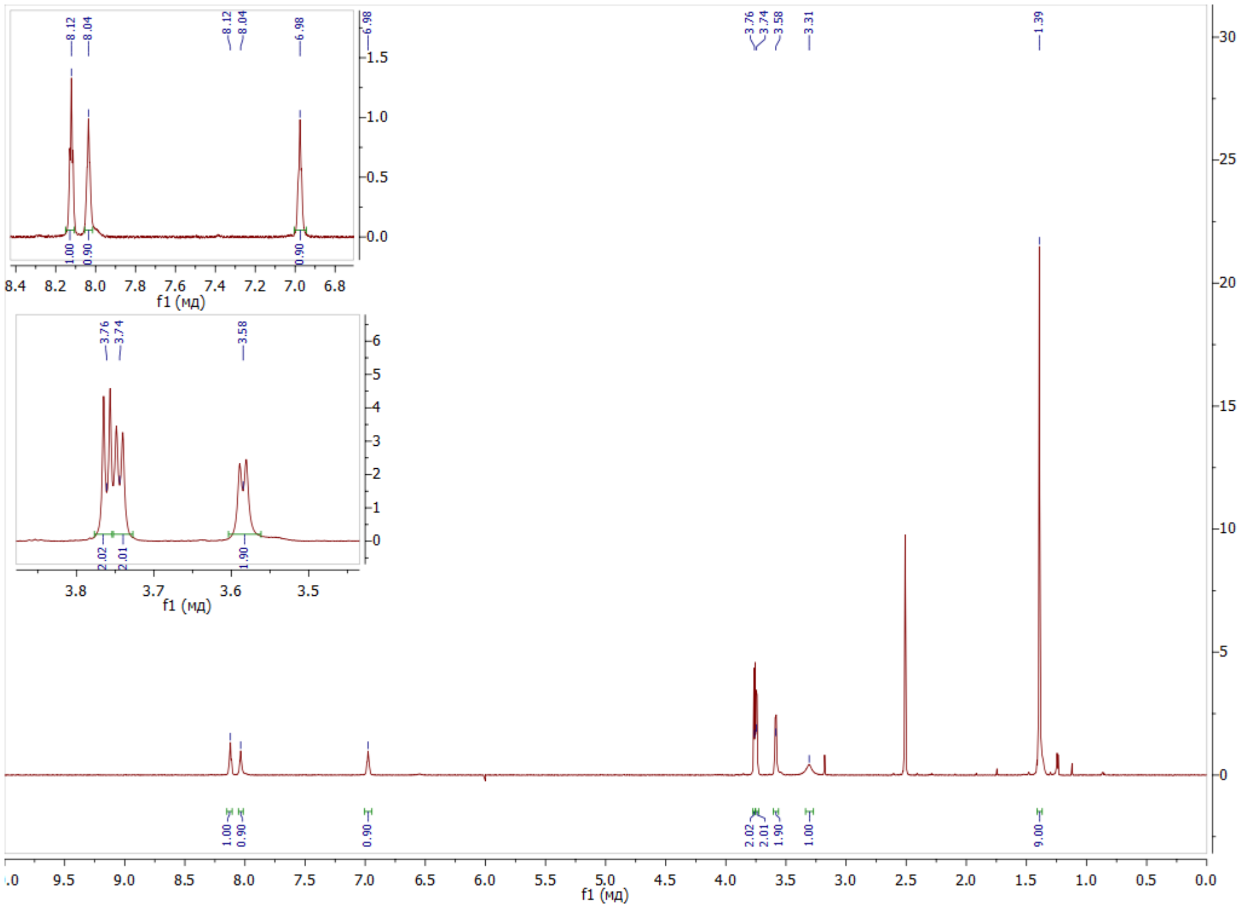

Figure S2-1. The 1H NMR spectrum of **Boc-Gly$_3$-OH**

2.2 Boc-Gly4-OH. HRMS, m/z: calculated for $C_{13}H_{22}N_4O_7$ [M + H]$^+$ 347.3450, found [M + H]$^+$ 347.3448. ^{1}H NMR (700 MHz, DMSO) δ 8.14(t, *J* = 5.60 Hz, 1H, NH), 8.07(t, *J* = 5.60 Hz, 1H, NH), 8.02 (t, *J* = 5.60 Hz, 1H, NH), 6.98 (t, *J* = 5.60 Hz, 0.9H, NH-Boc), 3.76 (d, *J* = 5.90 Hz, 2H, CH_2), 3.74 (d, *J* = 5.50 Hz, 2H, CH_2), 3.73 (d, *J* = 5.40 Hz, 2H, CH_2), 3.59 (d, *J* = 5.90 Hz, 2H, CH_2), 1.39 (s, 9H, Boc) ppm.

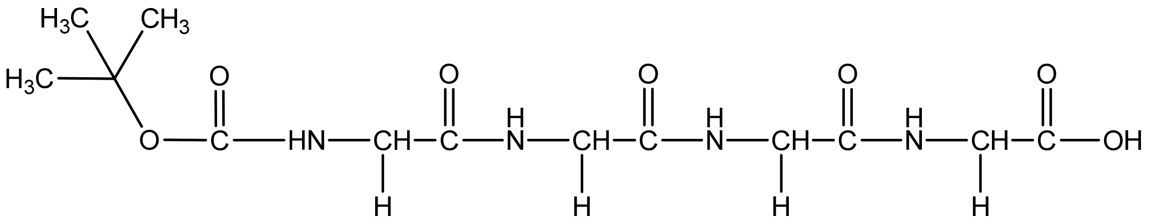

Chemical Formula: $C_{13}H_{22}N_4O_7$

Molecular Weight: 346,34

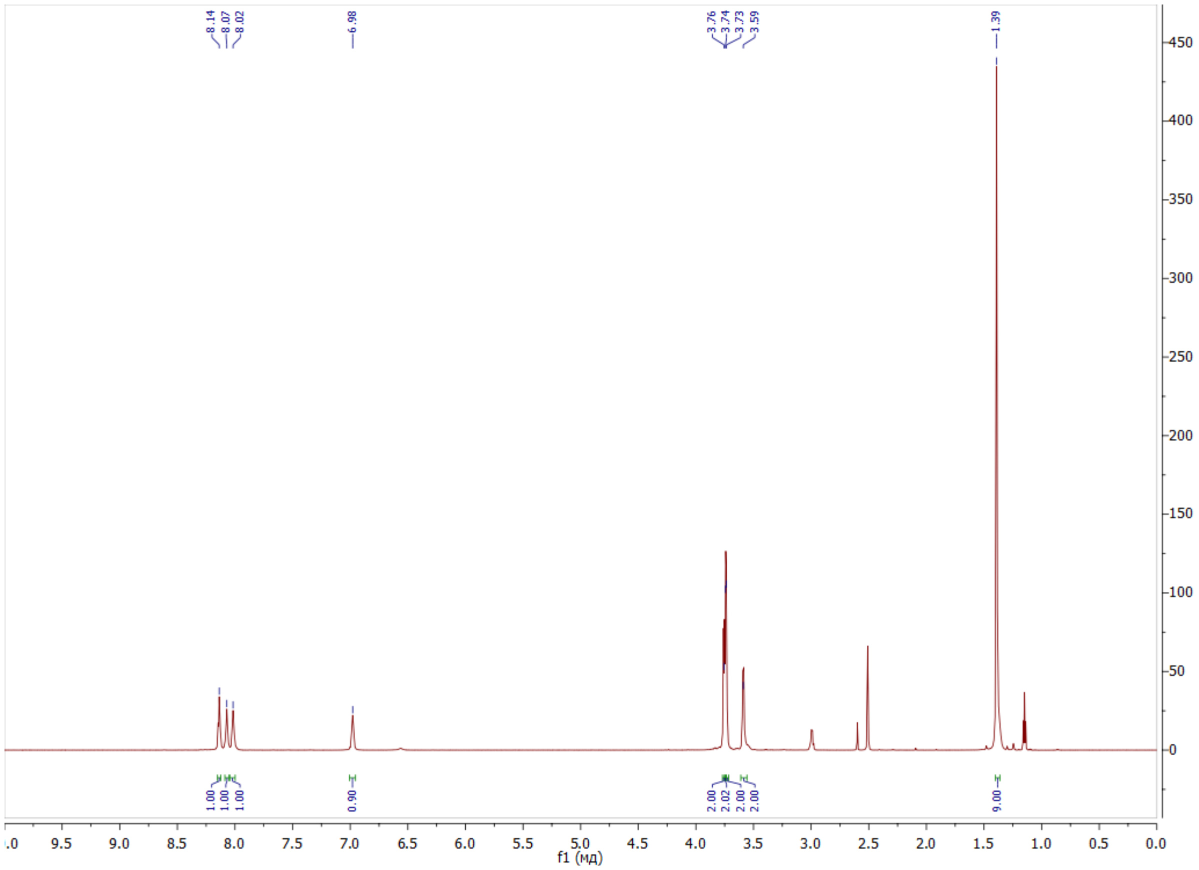

Figure S2-2. The 1H NMR spectrum of Boc-Gly4-OH

2.3 Boc-Gly$_5$-OH. HRMS, m/z: calculated for $C_{15}H_{25}N_5O_8$ [M + H]$^+$ 404.3964, found [M + H]$^+$ 404.3969. ^{1}H NMR (700 MHz, DMSO) δ 8.12 (br.sign., 2H, NH and NH), 8.08 (t, *J* = 5.63 Hz, 1H, NH), 8.03 t, *J* = 5.53 Hz, 1H, NH), 6.98 (t, *J* = 5.72 Hz, 0.9H, NH-Boc), 3.75 (br.sign., 8H, CH_2), 3.59 (d, *J* = 5.66 Hz, 2H, CH_2), 1.39 (s, 9H, Boc) ppm.

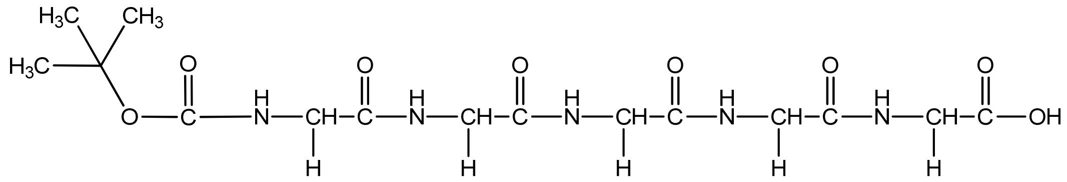

Chemical Formula: $C_{15}H_{25}N_5O_8$
Molecular Weight: 403,39

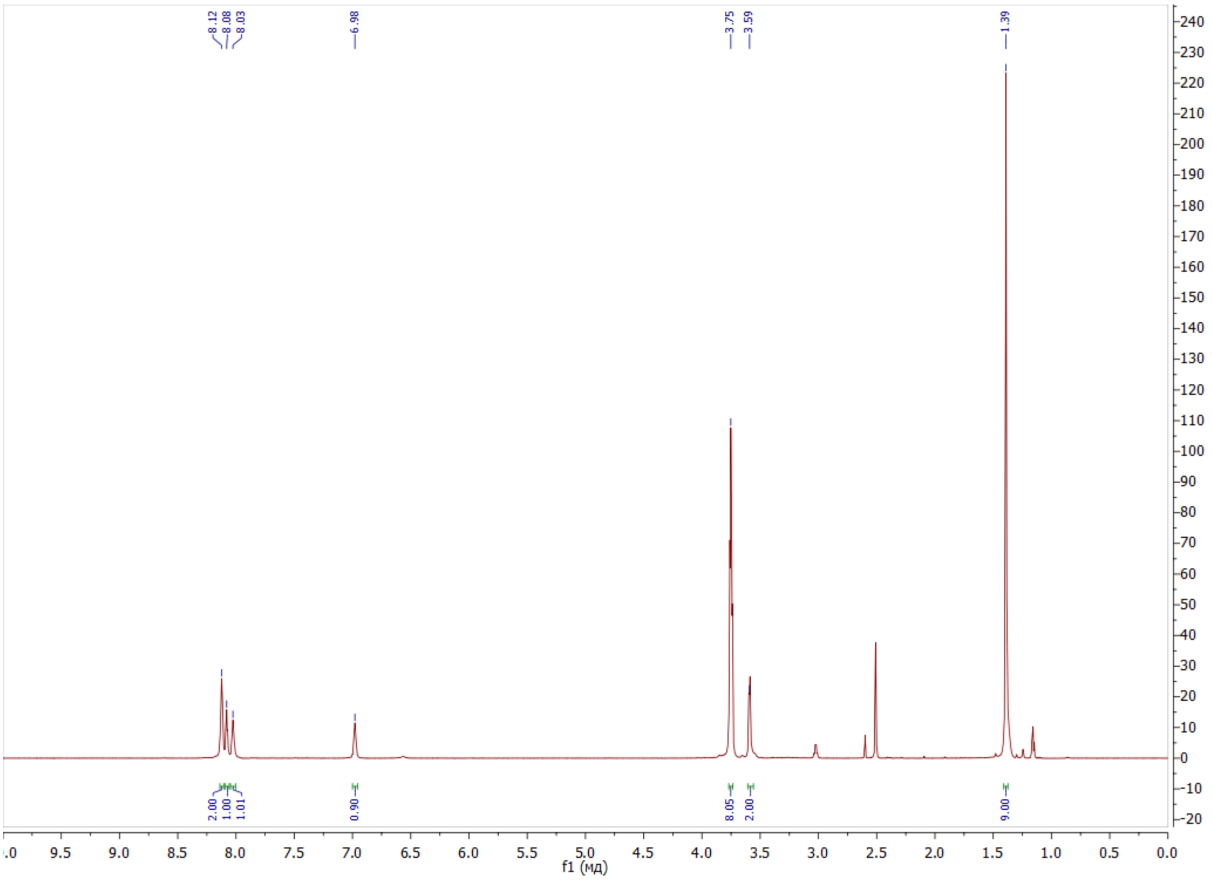

Figure S2-3. The 1H NMR spectrum of Boc-Gly$_5$-OH
